# The emerging implications of GLP-1 receptor agonists in radiation therapy

**DOI:** 10.1093/oncolo/oyag073

**Published:** 2026-03-03

**Authors:** Trenton Reinicke, Joshua T Dilworth

**Affiliations:** Department of Radiation Oncology, Oakland University William Beaumont School of Medicine, Rochester, MI 48309, United States; Department of Radiation Oncology, Corewell Health William Beaumont University Hospital, Royal Oak, MI 48073, United States

**Keywords:** radiation therapy, GLP-1 receptor agonists, cancer care, adaptive radiotherapy, weight loss

## Abstract

Glucagon-like peptide-1 receptor agonists (GLP-1 RAs) are increasingly prescribed for patients with diabetes, obesity, and cardiometabolic disease. This trend has led to a growing number of patients taking these medications while receiving cancer treatment, including radiation therapy (RT). The delivery of RT relies on weight stability and anatomic reproducibility, which may be influenced by GLP-1 RA-related weight loss, delayed gastric emptying, and associated side effects. While current research has largely focused on perioperative outcomes and metabolic effects, we discuss the impact that GLP-1 RA use may have on radiation treatment planning, the need for adaptive radiation techniques, and RT related toxicity. We propose strategies to support safe RT and highlight the need for consensus recommendations regarding the use of GLP-1 RAs in patients requiring cancer treatment.

Glucagon-like peptide-1 receptor agonists (GLP-1 RAs), like semaglutide and tirzepatide, are incretin-based metabolic therapies originally developed for the treatment of type 2 diabetes, but their use has recently expanded to obesity and cardiometabolic disease. In the United States, GLP-1 RAs are one of the fastest-growing classes of prescription medications, with their use rising from nearly 2.4% in 2010 to over 34% in 2024.^1^ Now, more than one in four adults with diabetes reports using GLP-1 RAs,^2^ and surprisingly, nearly 1 in 8 Americans, regardless of a diagnosis of diabetes, is taking this class of medication.^3^ With their increased use, there is a growing number of patients receiving GLP-1 RAs who require cancer care, including radiation therapy (RT). Despite this, there remains no consensus guidance for managing GLP-1 RAs during RT, a modality highly sensitive to weight stability and body habitus for safe and accurate treatment delivery. This represents an under-recognized but potentially clinically relevant gap in RT care.

Since cancer incidence and cardiometabolic comorbidities requiring the use of GLP-1 RA increase with age,^1^^,^^4^ an increasing number of adults will require RT while actively on GLP-1 RAs for non-oncologic indications. Unlike many cytotoxic or targeted systemic therapies that are frequently modified or discontinued at cancer diagnosis due to concerns regarding toxicity or immunosuppression, GLP-1 RAs are often continued in the absence of RT-specific guidance.

GLP-1 RAs’ effects on delayed gastric emptying, reduced appetite, and rapid weight loss raise practical concerns for RT planning. Patients receiving GLP-1 RAs may lose 5%-6% of their total body weight in 3 months.^5^ Since the time from simulation for RT treatment planning and the last dose of RT may span an equivalent time period (especially for those receiving conventionally fractionated regimens), these patients may undergo clinically meaningful changes in weight and anatomy throughout a course of RT. Target motion, imaging fidelity, and body habitus are all critical to accurate treatment planning and execution, but they are all subject to GLP-1 RAs effects. These distinctions among common GLP-1 RAs and their key pharmacologic characteristics, including expected weight-loss effects, are summarized in Table 1.

**Table 1:** Commonly used GLP-1 receptor agonists and dual incretin therapies: standard dosing and expected weight change in pivotal clinical trials.

Agent (trial)	Mechanism	Typical maintenance dose	Mean weight loss	Considerations during radiation therapy
**Semaglutide^19^**	GLP-1 receptor agonist	2.4 mg once-weekly SQ	∼12%-15% at 68 weeks	Rapid body habitus change may affect immobilization reproducibility
**Tirzepatide^20^**	Dual GIP/GLP-1 agonist	5 mg/10 mg/15 mg SQ weekly	∼15.0%-20.9% at 72 weeks (dose dependent)	Large mean weight reduction reported; potential need for adaptive planning
**Liraglutide^21^**	GLP-1 receptor agonist	3.0 mg SQ once daily	∼6%-8% at 56 weeks	Gradual weight change
**Dulaglutide^22^**	GLP-1 receptor agonist	1.5 mg/3.0 mg/4.5 mg SQ once weekly	−3.0 kg/-4.0 kg/-4.6 kg, respectively, by dose at 36 weeks	Common among oncology patients living with T2D

The standard maintenance doses used in pivotal clinical trials are depicted following recommended dose escalation. Cross-trial comparison is limited by differences in study populations, trial duration, and indications. Reported weight changes occurred under controlled trial conditions and may differ in patients undergoing cancer therapy or radiation treatment.

Abbreviations: GLP-1 RA, glucagon-like peptide-1 receptor agonist; GIP, glucose-dependent insulinotropic polypeptide; SQ, subcutaneous; T2D, type 2 diabetes.

While GLP-1 RA use has been studied among patients with certain forms of cancer, particularly in breast cancer, these studies have mainly focused on metabolic and weight-related outcomes, and not on the implications of using these drugs on the fidelity of RT delivery.^6^^,^^7^ Among the breast cancer cohorts in these studies, GLP-1 RA use was associated with modest but clinically meaningful weight loss, though no RT parameters such as dosimetric effects, treatment interruptions, or radiation-related effects were studied.^6^^,^^7^ Similarly, other studies among surgical patient populations on GLP-1 RAs in the perioperative setting exist in the literature; however, these outcomes focus on anesthesia-related risks and postoperative complications, rather than RT therapy.^8^^,^^9^ Thus, GLP-1 RA use during RT represents a largely unstudied, but expanding realm of cancer patient care for which further investigation is needed to reduce the uncertainty and heterogeneity in clinical practice. Even modest weight loss may alter patient anatomy, compromise immobilization reproducibility, and affect dose distribution to organs at risk.

As patients progress through RT, they may experience unintentional weight loss and multifactorial anatomic changes due to decreased caloric intake, dehydration, and treatment-related toxicities. The use of adaptive radiotherapy monitors anatomic changes that occur between simulation and treatment delivery, with weight loss frequently prompting repeat imaging or replanning.^10^^,^^11^^,^^12^ This framework may be helpful for patients on GLP-1 RAs, being an additional, independent, driver of anatomic change during RT. This concept has been studied using portal and electronic portal imaging device images.^10^ and with daily cone-beam CT to monitor anatomic deviations, such as tumor shrinkage or weight loss, which frequently require replanning.^11^^,^^12^ Particularly in head and neck RT, this surveillance method constituted replanning in roughly 10% of patients due mainly to weight loss.^11^^,^^12^ Toxicity overlap and a compounded nutritional risk are other concerns with concurrent GLP-1 RA use and RT, potentially increasing anorexia, dehydration, impaired wound healing, and treatment interruptions. The gastrointestinal (GI) side effects of GLP-1 RAs, like nausea, vomiting, decreased appetite, gastroparesis, and constipation, may mirror or augment RT-related GI toxicities, particularly during abdominal or pelvic irradiation with chemotherapy. In head and neck irradiation, where mucositis and dysphagia already complicate nutrition, the maintenance of weight stability is vital for maintaining mask fit and avoiding adaptive replanning. Although it is speculative, the established mucosal injury and delayed recovery of the GI barrier that occurs with RT^13^ could further amplify toxicity while on GLP-1 RAs, requiring close monitoring and further investigation into these interactions.

Given the various radiation-specific considerations that exist, GLP-1 RAs used during cancer treatment, such as androgen deprivation therapy (ADT) in prostate cancer, could further provide a therapeutic adjunct to the metabolic effects of ADT. Weight gain, insulin resistance, and cardiovascular risk are known to increase with ADT, thus GLP-1 RAs could mitigate some of these effects and offer a supportive-care strategy while on treatment. However, prospective data in oncology populations are needed.

The FDA Adverse Event Reporting System (FAERS), a voluntary adverse-event reporting system, has reported disproportionality signals for various cancers, including thyroid cancer with several GLP-1 RAs and tirzepatide.^14^ It is important to note that FAERS analyses can be limited by reporting bias and therefore cannot establish causality. Findings across various epidemiologic trials investigating cancer outcomes with GLP-1 RA use have been inconsistent. Thus, FAERS reports can be viewed as signals warranting further investigation and surveillance as these agents become more prominent in patients receiving cancer care.

The known delayed gastric emptying with GLP-1 RAs raises questions regarding potential interactions with oral systemic cancer therapies through altered absorption, as examined in a 2024 systematic review of injected GLP-1 RAs used alongside oral agents like warfarin, angiotensin-converting enzyme inhibitors, and statins.^15^ This study described a delayed time to peak concentration and modest reductions in peak drug levels while using GLP-1 RAs with certain oral medications, yet the total drug exposure remained typically unchanged.^15^ It is important to note that this study did not directly address oral cancer medications given in combination with injected GLP-1 RAs. Taken together, while most oral therapies are unlikely to require routine adjustment, it’s important to maintain awareness when GLP-1 RAs are used along with oral systemic cancer agents, where the timing of drug exposure is important, given the lack of literature describing their interactions.

To our knowledge, major oncology and radiation societies, including the American Society for Radiation Oncology (ASTRO) and the National Comprehensive Cancer Network (NCCN) do not currently guide GLP-1 RA management during or in the peri-RT period,^16^^,^^17^ resulting in a wide variation among RT planning, as these agents may be empirically held, continued with nutritional monitoring, or not recognized as relevant to RT planning. Given the heterogeneity in use of incretin-based therapies during RT, we propose the following practical considerations to raise awareness and promote consistency in clinical practice, without implying formal recommendations, pending further evidence:

- Baseline medication screening with documents such as referral or simulation paperwork explicitly inquiring about GLP-1 RA use.
- Risk stratification by treatment site and technique, where GLP-1 RA use may be more complicated, such as in head/neck RT with mask immobilization, abdominal/pelvic RT with possible mirrored or augmented GI toxicity, and with SBRT, where small margins exist.
- Weight and nutrition monitoring among patients continuing GLP-1 RA during RT, with adaptive planning if significant weight loss or body habitus changes occur. Optimal treatment may necessitate temporarily holding GLP-1 RA therapy during RT when weight loss is rapid or threatens immobilization reproducibility.
- Interdisciplinary coordination about any change to GLP-1 RA use, in consultation with endocrinology, primary care, nutrition, and other care teams.

In summary, GLP-1 RA use is rapidly expanding in clinical practice and in radiation oncology. It is essential to proactively evaluate their implications, especially given that medications with even more projected weight loss than current GLP-1 RAs are in clinical trials.^18^ Given the limited data that exists in this setting, studies to describe the prevalence of GLP-1 RA use among RT patients and their impact on RT efficacy and reproducibility are warranted. We feel that it would be prudent for large societies and interdisciplinary care teams to recognize GLP-1 RA use during cancer treatment as an emerging complexity requiring evidence-based recommendations. Establishing multidisciplinary awareness and guidance may help safeguard treatment quality.

## Data Availability

No new data were generated or analyzed in support of this research.
